# Structural issues affecting mixed methods studies in health research: a qualitative study

**DOI:** 10.1186/1471-2288-9-82

**Published:** 2009-12-09

**Authors:** Alicia O'Cathain, Jon Nicholl, Elizabeth Murphy

**Affiliations:** 1 Medical Care Research Unit, School of Health and Related Research, University of Sheffield, Regent Street, Sheffield, UK; 2 School of Sociology and Social Policy, Law and Social Sciences Building, University of Nottingham, Nottingham, UK

## Abstract

**Background:**

Health researchers undertake studies which combine qualitative and quantitative methods. Little attention has been paid to the structural issues affecting this mixed methods approach. We explored the facilitators and barriers to undertaking mixed methods studies in health research.

**Methods:**

Face-to-face semi-structured interviews with 20 researchers experienced in mixed methods research in health in the United Kingdom.

**Results:**

Structural facilitators for undertaking mixed methods studies included a perception that funding bodies promoted this approach, and the multidisciplinary constituency of some university departments. Structural barriers to exploiting the potential of these studies included a lack of education and training in mixed methods research, and a lack of templates for reporting mixed methods articles in peer-reviewed journals. The 'hierarchy of evidence' relating to effectiveness studies in health care research, with the randomised controlled trial as the gold standard, appeared to pervade the health research infrastructure. Thus integration of data and findings from qualitative and quantitative components of mixed methods studies, and dissemination of integrated outputs, tended to occur through serendipity and effort, further highlighting the presence of structural constraints. Researchers are agents who may also support current structures - journal reviewers and editors, and directors of postgraduate training courses - and thus have the ability to improve the structural support for exploiting the potential of mixed methods research.

**Conclusion:**

The environment for health research in the UK appears to be conducive to mixed methods research but not to exploiting the potential of this approach. Structural change, as well as change in researcher behaviour, will be necessary if researchers are to fully exploit the potential of using mixed methods research.

## Background

Mixed methods research has been defined as "integrating quantitative and qualitative data collection and analysis in a single study or a program of inquiry" [1]. There are definitions which include intra-method mixing, such as a survey with closed and open-ended questions, as well as inter-method mixing where two methods produce two sets of data. The definition used in this article requires that there are at least two methods in a single study, one qualitative - such as in depth interviews or focus groups - and the other quantitative - such as a survey or randomised controlled trial [2].

Mixed methods research has been of interest to health researchers for many years [3,4] and there is increasing interest in the topic within both health [5-7] and other research fields [8,9]. A variety of combinations of methods have been used in health research in the contexts of evaluating services, exploring health issues, and developing research instruments [10]. However, where methods have been combined with a justification of offering a more comprehensive picture, studies have been criticised for failing to integrate the data and findings from the qualitative and quantitative components to create a 'whole more than the sum of the parts' [11]. This is particularly noticeable at the publication stage of a study where it has been noted that it can be difficult to appreciate the interaction between parts of mixed methods studies because different components are published separately [12]. Indeed mixed methods studies in health can become less than the sum of their parts, with some components never published in journals, or different components published separately with little or no reference to each other so that any sense of their being mixed methods studies, and the possible benefits of this, are lost [13].

Prior to our study, some researchers had discussed structural issues affecting mixed methods research, although they tended to do this within articles or book chapters which focused on other issues rather than addressing structural facilitators and barriers centrally within their writing. Funding bodies were identified as affecting the existence of mixed methods studies - both as a hindrance and as a facilitator. In the 1990s, funding was seen as a practical constraint on combining methods in both social policy research and health services research because funding bodies had a preference for quantitative research [14] or for projects that were only quantitative or only qualitative [15]. Yet a conflicting view emerged later in the context of health policy research, when concern was expressed that funding bodies' desire for mixed methods studies might lead to inappropriate use of this approach [11]. Funding bodies were also held responsible for affecting the outputs of mixed methods studies. They were described as pressuring researchers to publish early from studies, resulting in qualitative and quantitative components being published separately [16]; or, setting short time frames for funding which might limit the amount of integration between qualitative and quantitative data and findings for some types of mixed methods studies [7].

Funding received most emphasis in the literature but other structural issues were discussed including education and training of researchers, peer-reviewed journals and academic career paths. It was noted that researchers may need training in both qualitative and quantitative methodologies to undertake mixed methods research. This was highlighted as a challenge [17] due to problems gaining training, particularly in the context of researchers on short term funding rather than established university posts, but also because people may have natural dispositions for one method rather than another [14]. However, it was also proposed that it may not be necessary to have an individual equipped to undertake both methodologies because researchers tend to work in teams where different team members can supply different methodological expertise [18]. Yet even in the context of a team, there is still a need for expertise in how best to combine methods, and integrate data and findings from different components. If this expertise exists then it is largely self taught because there are few training courses in mixed methods research [19]. Concerns were also expressed that journals may not accept mixed methods papers [20-22] and that academic promotion committees may not be able to engage with mixed methods research and may judge researchers through their own narrow methodological approach [20].

Prior to starting our study it appeared that structures could affect mixed methods research, although the literature showed that the effect was dependent on the research community - as described above, funding agencies were seen as a hindrance in one research community and a facilitator in another - and possibly the time period studied - for example, funding agencies may change their views of mixed methods research over time. Also, evidence of structural issues affecting mixed methods research was based on scholars' viewpoints rather than empirical study, and thus may not have identified the variety and complexity of perceptions of structural issues. Therefore, we undertook an empirical study to explore the structural facilitators and barriers to undertaking mixed methods research in health services research in the UK in the mid-2000s.

## Methods

We undertook a qualitative interview study of researchers who had worked on mixed methods projects. This was a component of a wider mixed methods study of how qualitative and quantitative methods are combined within health care research. It is necessary to describe the quantitative component of this wider mixed methods study before going on to describe the qualitative interview component, because interviewees were sampled from this quantitative component. The quantitative component involved identifying mixed methods studies funded by the health department of the government in England between 1994 and 2004. The proposals, reports and publications from these studies were obtained, and information was extracted on the quality of each study, the design types used, and the extent to which the study was assessed as exploiting the potential of mixed methods research[13]. This latter aspect was based on a subjective assessment of the extent to which researchers had used integration approaches identified in the mixed methods literature and relevant to that study, and an assessment of the attention paid to integration of data and findings from the qualitative and quantitative components within the set of publications emerging from that study[13]. These studies acted as our sampling frame for our qualitative interview study of researchers. Researchers' names were available from lists of applicants on proposals, lists of authors on reports, and lists of authors on articles. We undertook purposive sampling to include both qualitative and quantitative researchers, identifying this from the role researchers took within the proposal, report or journal articles. We also sampled to ensure that lead applicants, co-applicants and researchers employed for the study were included, and that different types of mixed methods studies were included, because researchers' views and experiences of mixed methods studies may differ by these characteristics. An effort was made to include researchers who had worked on studies which had been assessed as exploiting the potential of mixed methods research, or not, in the quantitative component of the study[13]. The purpose of this latter sampling strategy was to ensure that we could explore both facilitators and barriers to exploiting the potential of mixed methods studies.

When we selected individuals for interview, we sent them a letter asking for written informed consent to participate in an interview. All interviews were undertaken face-to-face in the interviewee's workplace. Our topic guide was focused on the project included in the quantitative component of our wider study, as well as mixed methods research generally. Our assumption was that our definition of mixed methods research was the same as that of the interviewees. We did not explicitly explore differences in definitions with interviewees but this did not arise as a problem because we had selected interviewees who had worked on studies which we had defined as mixed methods research. That is, our definition of mixed methods research was imposed on the data collection. By the 15th interview we noted that few new insights were emerging from interviews. By the time 20 interviews had been undertaken we felt that the available data were of sufficient breadth and depth to address our research question. One interviewer (AOC) undertook all the interviews in 2005. Interviews were recorded and transcribed verbatim. They lasted an hour on average, varying between 35 minutes and 90 minutes. Ethics approval was granted from the University of Sheffield Ethics Committee.

### Analysis

We read the interview transcripts to aid familiarisation with the data, and wrote a short summary of each transcript to maintain a case focus within the analysis. We developed a thematic framework based on the first ten transcripts. The themes were mainly descriptive, for example, when researchers explicitly or implicitly talked about publishing in peer-reviewed journals we organised this under a theme 'journals'. We applied the thematic framework to all transcripts, coding all parts to a theme or sub-theme using the computerised qualitative software package WinMAX [23]. We read the data extracts for each theme or sub-theme and undertook further coding within them to organise and understand the data. During this process we identified articles on cross discipline research as significant to the analysis [24,25]. These articles shaped our interpretation of the themes. We discuss here only the themes related to structural issues; other themes were identified such as the effect of team types on research outcomes, and reasons for undertaking mixed methods research.

## Results

We requested an interview with 22 researchers, 20 of whom agreed to be interviewed. The sample has been described in detail elsewhere [10]. Briefly, it included 11 quantitative and 9 qualitative researchers; researchers on 6 studies using a combination of randomised controlled trials and qualitative research, 8 on evaluation studies, 4 on studies using survey and fieldwork, and 3 on instrument development studies; 9 researchers were identified from studies assessed as exploiting the potential of mixed methods research.

### Funding and funding bodies

Funding bodies appeared to be powerful shapers of the type of research undertaken. Interviewees described how they monitored funding bodies' constituency and values. Although some funding bodies were identified as supporting mixed methods studies, and others as not, overall, some of the key funding bodies were seen as actively promoting mixed methods studies. This in turn had an impact on local work environments as different disciplines were employed or co-located in research units and departments, to facilitate mixed methods research and access to funding. Over time, as more mixed methods studies were undertaken in local environments, this approach was seen as the norm.

Funding bodies favouring mixed methods research was perceived as a facilitator for the existence of these studies, but not for exploiting the potential of this approach. The attraction of funding could encourage researchers' strategic use of mixed methods research, where the aim of combining methods was to gain funding rather than integrate methods for further insights [10]. Indeed there was no discussion of how funding bodies positively influenced the exploitation of mixed methods research by valuing integration between components of a study or publication of integrated outputs. In fact some interviewees argued that there was a cost to integration because it took time and did not seem possible within the timeframe of a two or three year study. Tight deadlines were identified as problematic particularly when using methods sequentially.

It may be that we're just very badly organised with our timetables. And you know you're always under pressure aren't you to do something in two years, maybe three years. But if you tried, if you said this is going to take us four years, I think your chances of getting funding would be pretty slim. So and yes, I do think that these mixed method projects are more complicated, they are more costly, more complicated, they're more costly so you get squeezed then in terms of time as well. R20

Publications were also a potential casualty of this time constraint. Researchers reported that there was time only to write what were perceived to be the main papers, or that the dispersal of a team at the end of a study resulted in separate publications by individual team members. Researchers described the pressure to move on to the next contract, or study, without fully exploiting the potential of their studies. This time constraint was identified as relevant to all studies, not simply mixed methods ones, but as possibly more of a problem for the latter because there was an increased likelihood of something going wrong with one of the methods and causing delay within a study timeframe.

### Education and training

The interviewees described using different combinations of methods, and different roles for methods, within their studies. They reported that they valued integration of the qualitative and quantitative components and discussed how this could occur at different stages of a study such as the design, sampling, and interpretation. However, there appeared to be gaps in researchers' knowledge because interviewees did not describe some of the integration techniques detailed in the literature [13]. Researchers themselves were sometimes aware of a knowledge gap around mixed methods research and wanted to see good examples of mixed methods studies, or identify the experts in this field. They cited the difficulty of undertaking integration when there were few or no examples of particular ways of combining methods in the literature, leaving them to devise new approaches 'on the job'.

At the moment I don't feel like there is that many, I suppose it's part of the same thing actually, examples of good quality mixed studies. You know I don't feel like I've got a feel for who is doing really good quality mixed methods studies and where. It's like if people want to know how to go and do good qualitative research, people often will direct them to particular people and at the moment I don't think ...and it's very, partly because people are publishing different studies in different places, and because they're not writing them up together. [...] It's really good when you do see them. R1

But I think that, perhaps another problem is the lack of sort of formalised methodology for doing this sort of work errrm...... You know you tend to have methodology in quantitative work, particularly in questionnaire design. You know it would be nice to have the processes linked, in some way save you having to make things up as you go along. R6

Knowledge about mixed methods research appeared to be gleaned from practical experience rather than studying it for a qualification, reading about it, or working with experts in it. Researchers reported building knowledge of exploiting the potential of mixed methods research over a series of studies, sometimes through making mistakes in previous studies.

I think in horror about how we first started off with our sort of ignorance and innocence about a lot of things, and it's been good to have the experience of learning how to do things better. I mean in the early studies I appreciate now that I really didn't incorporate very good qualitative methods and it would have added a lot more to the first studies had we sort of done that a bit, much better, in an actual 'organised from the start' way, planned in properly with proper sort of outcomes, and I think we've sort of better achieved this in this final study. R11

A barrier to exploiting the potential of mixed methods studies appeared to be the lack of educational infrastructure around this approach. Only one researcher discussed the influence of formal education and training on their mixed methods research. She had recently graduated from university and discussed how both qualitative and quantitative methods had been addressed in her training. However, the focus of her education was on research paradigms rather than training in mixed methods designs or synthesis of methods. As well as a lack of formal educational structures, it was apparent that there was also a lack of informal education through reading. This was highlighted by the way in which interviewees described the significance of particular books about qualitative research on their personal learning and on offering credibility to this approach, but did not discuss any books on mixed methods research even though this was the subject under discussion.

### Journals

The most discussed structural constraint on exploiting the potential of mixed methods research was peer-reviewed research journals. Researchers argued that if the journals which are respected in health care research - and these largely are the ones with high impact factors - do not accept qualitative research then this in turn directly affects the output of a study, in that the publication of the qualitative component may not be valued. Some journals which published predominantly qualitative articles were respected within the community, but the high impact factor journals mainly accepted quantitative studies. Journal impact factors were seen as very important in the context of national research assessment exercises, and because they affected the career prospects of researchers.

That ... the way the journals are set up, so like the [named journal] predominantly but not always, has traditionally taken quantitative papers, so it's hard to put qualitative research, or to mix those two things up, and it's getting the experience of the reviewers. I think it's a quantitative journal policy. I think that would have to change. R13

Even high impact factor journals which accepted qualitative papers were described as having strict word limits which shaped the type of qualitative paper which could be written. Word limits were also identified as prohibiting attempts at mixed methods articles. Some researchers described a 'catch-22' situation of having to attempt to keep within the required word limit of a journal whilst also giving all the information requested by journal reviewers. Word limits did not only affect mixed methods research, because debates were described within research teams about the other components of mixed methods studies, such as the health economics, and whether there was space to accommodate it within the 'main paper', with pleas not to "relegate the health economics to a second paper". R17

Journals were perceived as shaping study outputs because the templates were for qualitative articles or quantitative articles. Any movement outside these templates was seen as innovation which might put publication at risk. The result was the breaking up of studies into methodological pieces for publication, with priority given to the quantitative component.

But the problem is that the more innovative you are in that kind of co-analysis phase, the less publishable it is. So in terms of looking at, and as you mentioned, the closest we get is side by side papers, or the kind of stuff I've done which is you know the RCT didn't work and here is a little bit to say possibly why. They're kind of not very good qualitative analysis because it's a subsidiary kind of analysis but independently done. And the real kind of gap in the market methodologically I suspect is good methods of joining, along the whole research design process, so that for example we avoid the situation which I am normally in where there is an RCT and there is a bit of qualitative stuff tacked on. R14

Reviewers for journals were sometimes perceived as unable to engage with both components of a mixed methods article, leaving interviewees concerned that reviewers would dismiss the component they knew least about. Even though interviewees held negative perceptions of journal reviewers, some interviewees reported incidences of reviewers facilitating publications from mixed methods studies. For example one reviewer of an article based on the quantitative component of a study positively encouraged the inclusion of the qualitative component within the paper.

### The 'hierarchy of evidence'

A 'hierarchy of evidence' operates in health care research whereby some research evidence is considered more robust than others. This is applicable to studies of the effectiveness of health technologies such as drugs and services, where the randomised controlled trial is the gold standard. However in reality the effect of this hierarchy of evidence appeared to go beyond effectiveness studies by pervading the structures of health research. Some structures such as ethics committees were described as set up to deal with methods at the top of the effectiveness hierarchy. This could result in ethics committees either totally ignoring the qualitative component of a mixed methods study, or challenging the rigour of the qualitative component based on quality criteria for quantitative research. Additionally, interviewees described how the processes of ethics committees could not accommodate the way that the development of one method might be dependent upon the completion of another method in some mixed methods studies. This was described as problematic for instrument development studies where researchers had to return to ethics committees for further approval on a number of occasions throughout the study, causing delays to the smooth execution of mixed methods research. The 'hierarchy of evidence' also affected publications from mixed methods studies, with researchers placing more importance on publishing the randomised controlled trial than the qualitative component.

### The necessity of serendipity and effort

The norm defined by the structures appeared to have a powerful effect on researchers' ability to exploit the potential of mixed methods studies. Many of the researchers used the term 'luck' when talking about their experiences in mixed methods research - being 'lucky' to work within a good team, being 'lucky' to have a boss who supported qualitative research, or being 'lucky' to have a job. Luck was seen as playing a particularly important role in one study where the lack of respect of the lead researcher for the qualitative component remained throughout the study and yet surprisingly the qualitative component was published in a high ranking journal. Serendipity was necessary in exploiting the potential of this mixed methods study; the publication of the qualitative component relied upon the quantitative project leader being unavailable to influence decision making at a crucial stage.

As well as luck, there was much talk of the effort involved in both undertaking, and exploiting the potential of, mixed methods studies. Qualitative researchers talked about struggling and battling to convince colleagues of the worth of their methodology. The 'hierarchy of evidence' appeared to create a 'hierarchy of methods' which interviewees described as a tide which qualitative researchers had to swim against, because of the effort required to convince journal editors and team members of the worth of qualitative research, or value of integrating the qualitative and quantitative components of a study.

It took us a few years to get through, you know we were absolutely convinced we were doing the right thing, but then no one would listen to us. So we did have to battle a bit. R16

So those are the sorts of circumstances I would feel I was sort of slightly fighting a battle with my colleagues, who don't really understand. I mean you know, you think people are going to understand qualitative research but actually then you suddenly realise that they don't, they don't actually understand what it's all about. [...] I've got one particular colleague on this trial, who shall remain nameless, who is very strong on the quantitative side, who grudgingly has come to accept that qualitative research might have some role as a sort of preliminary to other things, but I can't really shift his main thinking, which is that science is really about [...] hypotheses and testing hypotheses rigorously. R9

### Researchers as agents within structures

The environment is not a passive external force but one which is supported by individual researchers who sit on the editorial boards of journals and the commissioning boards of funders, and who review articles and proposals for these bodies. There was some evidence within the interviews of the influence of individual researchers who exercised their power within these roles to facilitate the existence and exploitation of mixed methods studies, for example encouraging the qualitative component of a study into existence, influencing a quantitative researcher not to reduce the size of a qualitative chapter in a draft report, or offering a qualitative researcher reassurance about the quality and usefulness of their contribution.

and they have social scientists [...] So you know it's got in there, you know qualitative researchers have sort of infiltrated these large funders, and I think that's a good thing. R3

It's very kind of gratifying to see that the reviewers' also commented that the [qualitative] chapters were interesting. R4

Interviewees noted that the research climate had changed over time, becoming more favourable both towards qualitative and mixed methods research. The research environment, within local departments and the general research community, was described as continuing to improve, for example with the development of groups of qualitative researchers rather than lone qualitative researchers, which allowed them to assert their approach to research within mixed methods studies.

And qualitative research in the [research unit] is I think much, much stronger now than it was at that time, and I was saying, I was actually talking to somebody about [the mixed methods project] and I think in some ways the opportunity I had with [the mixed methods project] in terms of qualitative research I probably would not have now, if I'd been at the same point in my career, because there are people who are really strong in terms of qualitative research, and there are other people feeding into sort of trials in qualitative research, and I suppose not just qualitative research, but that sort of, the more sort of social constructionist perspective as well, whereas that wasn't there at the time. And that really wasn't, I mean that whole collaboration, there just actually weren't people doing qualitative research in a lot of the institutions. R1

## Discussion

Researchers perceived that the research infrastructure in the UK in the mid-2000s facilitated the use of mixed methods studies in health care research but hindered exploitation of this approach. Structural facilitators for undertaking mixed methods research were a perception that funding bodies promoted this approach, and the multidisciplinary constituency of some university departments. Although funding bodies were perceived as facilitators for the use of mixed methods research, they appeared to do nothing to promote integration of qualitative and quantitative components and thus help to exploit the potential of this approach. Another key structural barrier to exploiting the potential of these studies was a lack of education and training in undertaking mixed methods research, for example the study of design typologies and integration techniques. Researchers' education was experiential rather than through formal postgraduate courses or informal reading of key methodological texts. The opportunity for informal education may have increased since this research was undertaken because so many books have been published in recent years, but the lack of formal education in mixed methods research is unlikely to have changed, and is by no means unique to health research or the UK [26]. Peer-reviewed journals, with their perceived orientation towards either qualitative or quantitative research, and their limitations on article length, were not seen as offering opportunity for publishing mixed methods articles. The 'templates' offered were for either a qualitative paper or a quantitative paper [27] leaving researchers feeling that they would be taking the risk of jeopardising publication if they wrote a mixed methods paper. The word limit of some journals was problematic for all researchers but some of them found ways around this by writing a series of papers published in sequence drawing on different components of a study but paying attention to integration of qualitative and quantitative findings within some of these papers [13].

Structural issues for mixed methods research have been identified in other research communities. A study of mixed methods journal articles in social research identified that, even when qualitative and quantitative findings were presented in the same article, they were rarely integrated [28]. A qualitative study of social researchers identified that they considered it challenging to integrate the qualitative and quantitative components of a study [29]. Barriers included those intrinsic to research methods, such as differing time lines of methods leading to separate analysis and publication; those related to the skills and preferences of researchers, such as different team members undertaking different components of a study; and structural barriers, such as different journals emphasising either qualitative or quantitative research. Structural barriers have also been discussed in the related context of cross discipline research [24,25,30]. These were very similar to the issues found in this study and included funding, education, journals, and academic careers. Discussions about career paths have been mixed, identified as problematic for cross discipline researchers because career paths exist for single disciplines only [25] or as a facilitator for academic careers [31]. The necessity of effort has been identified elsewhere too, where the lack of infrastructure support has led to a call for researchers to "be courageous risk-takers" and not to be discouraged by the lack of incentives [25].

### Limitations

The study took place in one research community in one country when structural facilitators and barriers may differ by context. However, our findings add to a growing literature from other research communities [29] in other countries [31] showing how the research infrastructure shapes studies and their outputs. We found only facilitators to undertaking mixed methods research in health care research in the UK. This may have been because we only interviewed people who had managed to undertake mixed methods studies.

Time has elapsed since this fieldwork was undertaken in 2005. Since then, there has been an annual mixed methods conference in the UK, a new journal devoted to mixed methods research - the Journal of Mixed Methods Research - and a large increase in books and articles written about this methodological approach. It is important to ask whether the findings of the study have temporal transferability to 2009 [32]. We have disseminated this work widely through workshops and seminars in the UK and Ireland and found it still to be highly relevant.

We have previously expressed concern that mixed methods studies in health tend to be broken up into their methodological components for publication in peer reviewed articles [13]. Yet we have presented here only the findings from the qualitative component of a mixed methods study. In defence, we have written five articles from this mixed methods study: two based only on the quantitative data, one based on both the qualitative and quantitative component, one based on the qualitative component which used data from the quantitative study, and now this one which is based on the qualitative component only but displays the role of the quantitative component in sampling interviewees. Our argument is that mixed methods studies should yield at least some papers which focus on a conversation or integration between the qualitative and quantitative components, and we have done this.

### Implications

The environment for health research in the UK appears to be conducive to mixed methods research but not necessarily to the exploitation of this approach. There is a need to change structures such as how researchers are educated and how journals invite and offer space to mixed methods papers. Changes to curricula of postgraduate courses may involve discrete additions of sessions about mixed methods research to modules which deal separately with qualitative and quantitative methodology, or a more radical upheaval of having research methodology modules which simultaneously cover the different aspects of both methodologies within a mixed methodological framework [33]. Researchers are agents who may also support current structures - journal reviewers and editors, and directors of postgraduate training courses - and thus have the ability to improve the structural support for exploiting the potential of mixed methods research.

## Conclusion

The external environment for health research in the UK appears to be conducive to mixed methods research but not to exploiting the potential of this approach. Structural change, as well as change in researcher behaviour, will be necessary if researchers are to fully exploit the potential of using mixed methods research.

## Competing interests

The authors declare that they have no competing interests.

## Authors' contributions

AOC conceived the study, collected and analysed the data, and wrote the first draft of the paper. EM and JN contributed to the design and analysis of the study and commented on drafts of the paper. All authors read and approved the final manuscript.

## Pre-publication history

The pre-publication history for this paper can be accessed here:

http://www.biomedcentral.com/1471-2288/9/82/prepub
